# Caribbean Islands facilitated the colonization of Western Hemisphere barn owls, yet experienced rampant extinction

**DOI:** 10.1098/rspb.2026.1022

**Published:** 2026-10-21

**Authors:** Min Zhao, David W. Steadman, Michelle J. LeFebvre, Robert P. Guralnick, Joshua R. Doby, Julie M. Allen, Jessica A. Oswald

**Affiliations:** ^1^ Florida Museum of Natural History, University of Florida, Gainesville, FL 32611, USA; ^2^ Department of Biological Sciences, Virginia Tech, Blacksburg, VA 24061, USA; ^3^ U.S. Fish and Wildlife Service, National Fish and Wildlife Forensics Laboratory, Ashland, OR 97520, USA

**Keywords:** phylogenomics, biogeography, *Tyto furcata*, extinction, barn owls

## Abstract

Island biodiversity reflects multiple evolutionary roles, encompassing lineages derived from nearby continents, relict taxa and, in limited cases, sources of continental diversity. Reconstructing the biogeographic history and island-mainland diversification dynamics requires an integrated understanding of speciation, extinction and dispersal processes to and from islands. Extensive Holocene extinctions may obscure patterns of diversity and connectivity. Tytonidae (barn and bay owls) is a bird group with a worldwide distribution and numerous Quaternary extinctions. Here, we use ultraconserved elements and mitogenomes extracted from tissues, historical museum specimens and fossils to reconstruct phylogenetic relationships and colonization pathways, with a focus on Western Hemisphere taxa. We also summarize extinct *Tyto* diversity to evaluate Quaternary diversity trends. Our results align with previous studies in supporting an Eastern origin for Western Hemisphere *Tyto*. We found that the Caribbean was, and still is, the centre of *Tyto* diversity in the Western Hemisphere, although nearly all Caribbean *Tyto* (and their mammalian prey) are now extinct. Unexpectedly, we identified two independent *Tyto* colonization events of the Galápagos. Our study provides strong evidence that islands can serve as sources of diversity for some continental lineages, as well as museums of diversity that harbour unique lineages prone to disturbance-driven extinction.

## 1. Introduction

Islands have long been recognized as ‘natural laboratories’ because their isolation creates tractable settings for studying evolutionary processes (reviewed by Whittaker *et al.* [1]). Island biotas often exhibit exceptionally distinct evolutionary and phenotypic diversity (e.g. [2]). In fact, many islands act as ‘museums’ of diversity as they are the last respites of once more widespread and diverse clades (e.g. [3–5]). This diversity has often been thought to be sourced from nearby continents, an idea that is the foundation of island biogeography. However, evidence indicates that speciation, extinction and migration to and from islands are more complex and that, in some cases, islands serve as the source of continental diversity (e.g. [6–8]).

Understanding the history and patterns of biotic diversification on islands has been made challenging by anthropogenic processes, particularly during the Holocene [9]. Human presence has led to extinctions, extirpations and translocations of species on islands worldwide [10,11]. Nearly half of all endemic insular bird species present in 1500 Common Era (CE) are now either extinct or threatened [12]. These extinctions diminish phylogenetic, functional and morphological diversity on islands and disrupt trophic networks [13]. Human-driven habitat changes and introduced species (e.g. rodents) have also enabled non-native species to ‘naturally’ disperse across islands [14]. Consequently, human impacts often confound or entirely erase biogeographic and evolutionary patterns if only extant taxa are examined [15–17].

The barn and bay owls (Tytonidae) have a nearly worldwide distribution, including many islands. Tytonidae comprises 20 extant species (18 *Tyto* and two *Phodilus*), of which nine are monotypic and 11 are polytypic, together encompassing 62 recognized taxa. Within *Tyto*, over half of the species (56%) and subspecies (53%) occur only on islands, including several IUCN-listed threatened species [18]. Most Tytonidae species are distributed in the Eastern Hemisphere, with only two extant *Tyto* species in the Western Hemisphere (*T. furcata* and *T. glaucops*). Owing in part to the extensive distribution of common barn owls (*T. alba sensu lato*) and substantial variation in their plumage, size and vocalizations [19,20], diagnosing evolutionary lineages has been challenging. *Tyto furcata*, the American barn owl, was only recently (2024) split from *T. alba*, with *T. alba sensu stricto* in Europe and Africa and *T. javanica* in Asia, Australasia and southwest Pacific [21]. For *T. furcata*, 11 subspecies are recognized, mainly based on plumage and biogeography; among these, six are endemic to islands. Some also consider the Lesser Antillean *T. f. insularis*/*nigrescens* and Galápagos *T. f. punctatissima* as species (e.g. [22,23]), although genetic data are needed to test these hypotheses.

Late Quaternary fossil records of *Tyto* portray major losses of island diversity, especially in the Caribbean, where many extinct *Tyto* are thought to have fed on likewise extinct, endemic mammals (e.g. [24–26]). Therefore, Tytonidae offers a unique system for studying diversification and colonization across continents and islands through time, and for assessing human impacts and environmental changes on their diversity, survivorship and range expansion.

Here, we investigate the evolutionary relationships, biogeographic patterns and global diversity losses of Tytonidae, focusing on *Tyto* in the Western Hemisphere through comprehensive sampling of ultraconserved elements (UCEs) and mitochondrial genomes (mitogenomes) from tissues, toe pads and fossils, with an emphasis on the Caribbean.

## 2. Material and methods

### (a) Taxon sampling

We collected tissues, ribs (≥25 years old), toe pads from museum study skins (collected from 1876 to 1995), and samples from *T. furcata* fossils (supplementary material, table S1). For samples representing *T. furcata*, we assigned them to subspecies following eBird/Clements Checklist v.2024 [18]. We attempted to collect DNA from five fossil samples of the giant extinct *T. ostologa* and three fossil samples of the giant extinct *T. pollens* (supplementary material, table S2). For extant species, we did not sample the extremely range-restricted *T. almae* and *T. nigrobrunnea* from the Maluku Islands of Indonesia and *T. prigoginei* from the Itombwe Mountains of the Democratic Republic of the Congo.

### (b) DNA extractions and library preparation

A single fossil sample was processed per day, with negative controls, following protocols in Oswald *et al.* [27]. DNA from ribs was extracted using the same protocol as for the fossil samples. We used a modified protocol from Soares *et al.* [28] for toe pad samples, and for tissues (performed in a lab separate from the ancient/historical samples), we used the Qiagen DNeasy Blood & Tissue Kit following the manufacturer's protocols. DNA libraries were produced using IDT xGen^TM^ library prep kits. For modern and historical samples, mitochondrial baits (Arbor 303008.v5, developed based on *T. alba*; GenBank accession EU410491) were mixed with UCE baits (1:250 µl; Arbor myBaits UCE Tetrapods 5Kv2) to increase the recovery of both mitogenomes and UCEs. Libraries from fossil samples were double-enriched following the myBaits high-sensitivity protocol with mitochondrial baits. For detailed steps of DNA extraction and target enrichment, see the supplementary material.

### (c) Extracting ultraconserved elements from modern and historical samples

We pre-processed the raw reads using fastp v.0.23.4 [29] to remove adapters and low-quality reads. We used SPAdes v.4.0.0 [30] to assemble UCE contigs following settings described in Zhao *et al*. [31]. We extracted UCEs (and upstream and downstream 500 bp around the conservative core) from the SPAdes assemblies using the UCE 5K probe set in PHYLUCE [32]. Similarly, we also extracted UCEs from published genome assemblies (supplementary material, table S1).

### (d) Extracting mitogenome from modern and historical samples

We applied the eager pipeline [33] (nf-core/eager v.2.5.2 [34]) using Nextflow v.22.10.6 [35] to extract mitogenome from target enrichment raw reads by read mapping to a *T. alba* mitogenome (GenBank accession MZ318036) using Bowtie2 [36]. mapDamage [37] was used to assess potential ancient DNA (aDNA) damage. See the supplementary material for additional eager settings. We used SAMtools v.1.20 [38] to generate a consensus sequence from the mapped reads with a minimum mapping quality of 30. For each sample, we aligned its consensus sequence to the reference and transferred the mitogenome annotations using Geneious v.11.0.20.1.

We also downloaded published mitogenomes from the National Center for Biotechnology Information (NCBI) GenBank (supplementary material, table S1) and Salter *et al.* [39]. For *T. novaehollandiae*, we downloaded mitochondrial cytochrome *b* (Cytb) sequences available on GenBank, since our sample did not yield usable data.

### (e) Phylogenetic inference

We found that most samples yielded substantial UCE loci, though some historical samples produced few loci with high missing data (supplementary material, table S3). Therefore, we first assembled a UCE dataset with good-quality samples (>1500 loci) and aligned the UCEs using MAFFT 7 [40] implemented in PHYLUCE. We then estimated a maximum likelihood (ML) tree (T_0_) in IQ-TREE v.2.2.2 [41], using ModelFinder [42] to select the best-fit partitioned models and 1000 ultrafast bootstraps to evaluate branch support. Next, we collapsed the branches in tree T_0_ with support below 70. Lastly, we constructed a dataset containing samples with >200 loci and estimated a phylogeny (T_1_) in IQ-TREE2, using the collapsed tree T_0_ as a topological constraint. Samples with <200 UCE loci were discarded owing to their poor phylogenetic performance.

We used weighted ASTRAL [43] to estimate two species trees under the multispecies coalescent model, respectively, from gene trees estimated by IQ-TREE2 from: (i) the UCE dataset of samples with >1500 loci and (ii) the UCE dataset containing samples with >200 loci. We also estimated species trees from the two UCE datasets using a site-based coalescent method SVDquartets [44] via PAUP* v4 [45], based on 1000 bootstrap replicates using 500 000 random quartets.

We aligned mitogenomes using MAFFT v.7.520 [40] and examined the alignment by eye in Geneious. We trimmed most of the D-loop before tree estimation because these regions have large gaps, in part owing to high divergence and missing data. We estimated a mitogenome tree in IQ-TREE2, using ModelFinder to test for the best-fit substitution model and 1000 ultrafast bootstrap replicates.

### (f) Divergence time estimation

We used BEAST v.2.7.7 [46] to estimate divergence times from the mitogenome dataset under the calibrated Yule model. We set three groups as monophyletic (Tytonidae, Strigidae and Strigiformes) and applied fossil calibrations for crown Tytonidae (30.9–37.5 million years ago (Ma)), crown Strigidae (20.0–23.03 Ma) and crown Strigiformes (37.5–61.7 Ma), each under a uniform distribution. See the supplementary material for fossil justifications.

We used GTR+I+G as the site substitution model, and the optimized relaxed clock model [47] to estimate lineage-specific rate variation. We ran four independent BEAST runs on CIPRES Science Gateway [48], each with 50 000 000 generations and sampled every 1000 generations. We combined the tree files from the four runs using LogCombiner with a 10% burn-in and summarized a maximum clade credibility tree using TreeAnnotator. Convergence of chains and effective sample sizes were assessed in Tracer v.1.7.1 [49].

### (g) Biogeographical reconstruction

We used the time-calibrated mitogenome tree to reconstruct ancestral range with BioGeoBEARS v.1.1.3 [50] by performing two analyses: one focusing on global patterns and the other focusing on Western Hemisphere taxa.

First, we pruned the tree to keep only one sample representing each Tytonidae species and assigned each species to one or more of the four areas (Americas, Africa, Eurasia and Oceania). We tested DEC, DIVALIKE, BAYAREA, with and without the long-distance dispersal parameter (+j), in BioGeoBEARS because *Tyto* colonized the Americas from the Eastern Hemisphere likely via trans-Atlantic dispersal, based on both our results (see below) and previous studies [51,52]. Though we recognize taphonomic bias in tropical regions, *Tyto* fossils have not been found in any pre-Quaternary sites in the Americas, whether on continents or islands. While absence of evidence is not evidence of absence, pre-Quaternary bird fossils are extensive on the American continents, though not in the Caribbean. Given this and previous biogeographical findings [52], we performed a stratified analysis by disallowing ‘Americas’ to appear as an ancestral state before 2.6 Ma.

Second, we performed another BioGeoBEARS analysis by including only *T. alba* and Western Hemisphere taxa. We assigned *T. alba* to the Eastern Hemisphere and other taxa to six regions (North America, Central and South America, The Bahamas, Greater Antilles, Lesser Antilles and Galápagos). All subspecies of *T. furcata* were included only once, except for *T. f. pratincola* as it occurs in three regions and was therefore represented by three tips. A similar stratification was applied to disallow any of the Western Hemisphere regions to appear before 2.6 Ma.

### (h) Summarizing Quaternary *Tyto* extinctions on islands

To place current diversity within the broader context of known extinct forms, we reviewed palaeontological and archaeological publications that included descriptions or the recovery of extinct *Tyto* materials, and summarized their historical range, taxonomy, possible last occurrence and presumed primary prey. While our focus herein is largely on the Western Hemisphere, global *Tyto* taxa were reviewed to assess variability of extinction patterns in Tytonidae.

## 3. Results

### (a) Taxon sampling and sequence data summary

We sampled 15 *Tyto* and two *Phodilus* species (figure 1), representing all extant Tytonidae except for three Eastern Hemisphere species (see Material and methods). Within *T. furcata*, we sampled all 11 currently recognized subspecies across its natural range, including fossil samples from the Caribbean (figure 2). None of the extinct *T. ostologa* and *T*. *pollens* samples (supplementary material, table S2) yielded aDNA.

**Figure 1.**
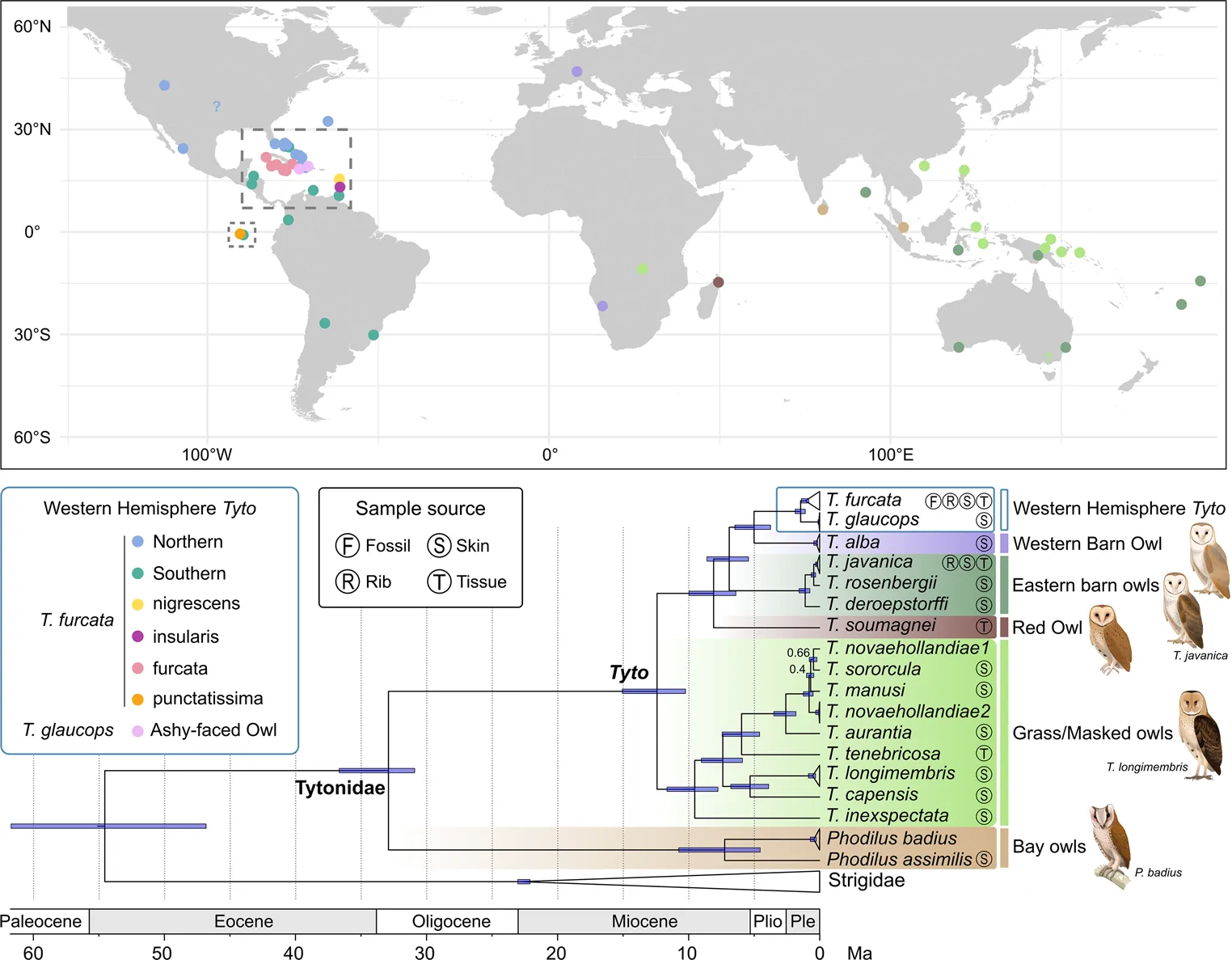
Global sampling localities and the time-calibrated mitogenome tree. Points on the map are colour-coded based on clades in phylogeny. The exact locations for two *T. novaehollandiae* samples (GenBank) and one *T. furcata* sample (NCBI genome) were not provided and are thus shown as question marks (Australia and USA, respectively). The two dashed boxes outline the Caribbean and Galápagos (see figure 2). We collapsed nodes in the time tree (see tree file in the data repository), to keep one tip representing each species, except for *T. novaehollandiae* owing to paraphyly (*novaehollandiae1* from Papua New Guinea, and *novaehollandiae2* from Australia). We marked the sample source near the tip label only for samples collected by our study. Values near nodes show posterior probabilities (full support omitted). Node bars show the time interval that contains 95% highest posterior density (HPD). Owl illustrations by Qiuyang Zheng.

**Figure 2.**
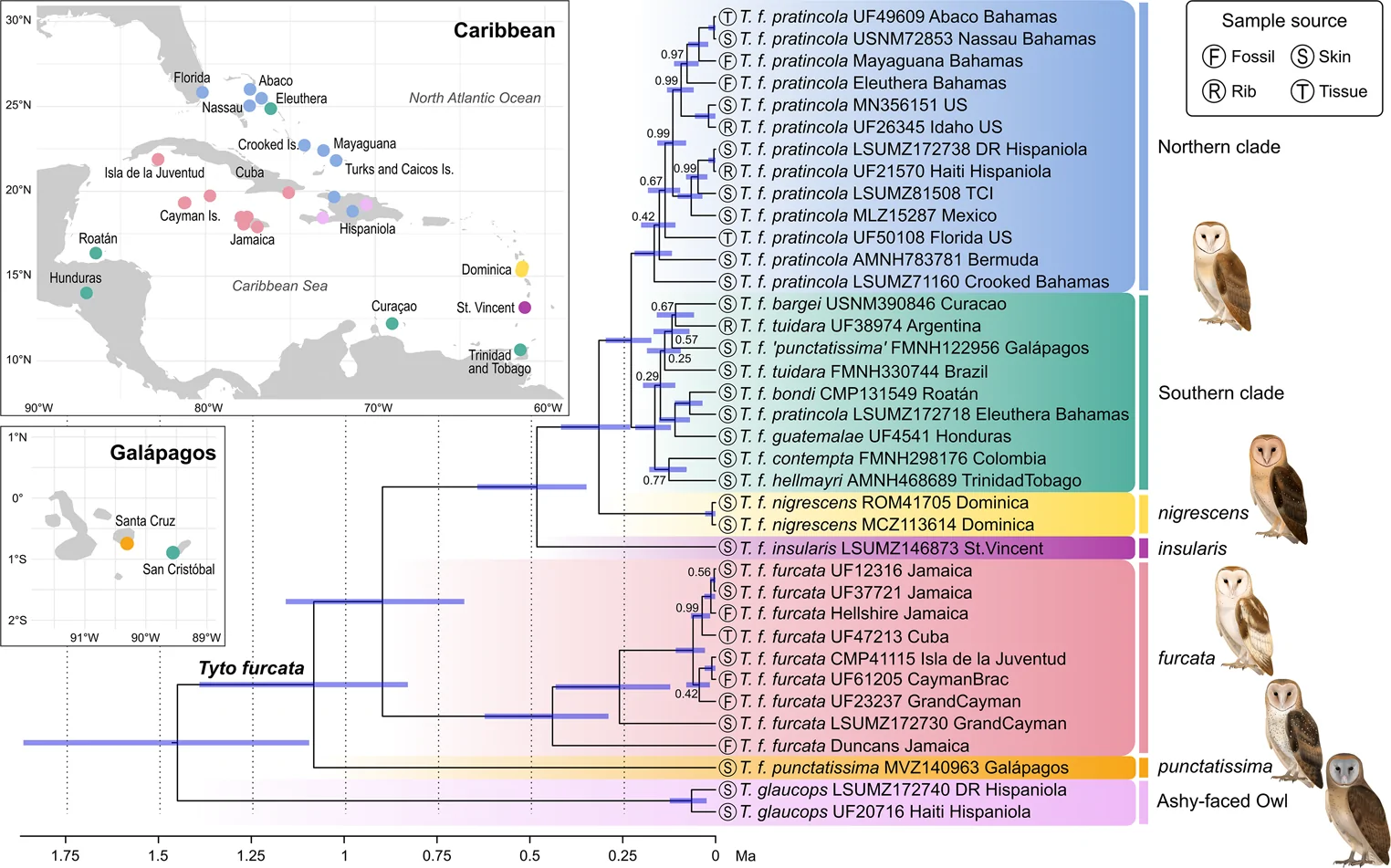
Sampling locality in the Caribbean and Galápagos and the pruned, time-calibrated mitogenome tree. Values near nodes show posterior probabilities (full support omitted). Node bars show the time interval that contains 95% HPD. Full institutional names corresponding to museum codes are provided in table S1 in the supplementary material. Owl illustrations by Qiuyang Zheng.

After data quality filtering, we included 42 samples in the final UCE dataset (supplementary material, table S1), with an average of 3322 loci per sample and over 80% of the samples with >1500 loci (supplementary material, table S3). Among them, 32 samples were produced by our study (81% from historical samples (toe pads and ribs)). Our mitogenome dataset included 68 samples, among which 52 were mitogenomes produced by our study (88.5% from toe pads, ribs and fossils).

### (b) Radiocarbon dates

*Tyto furcata* fossils that successfully yielded organics were from the Cayman Islands, Jamaica and The Bahamas (supplementary material, table S2). Radiocarbon dates from these sites (supplementary material, table S4) were less than 220 ^14^C years Before Present (BP) with the oldest material from Jamaica (180–220 years BP). As such, sequences from these fossils lack the DNA damage patterns typical of aDNA (supplementary material, figure S1). *Tyto pollens* or *T. ostologa* fossils were not radiocarbon dated as they did not yield target DNA and to limit destructive sampling.

### (c) Phylogenetic relationships

Our UCE phylogeny based on good-quality samples (>1500 loci; T_0_) recovered well-resolved and strongly supported major clades (supplementary material, figure S2). Incorporating lower-quality samples (>200 but <1500 loci) in tree T_1_ did not alter the major clade topology, and these samples grouped with their expected clade based on locality (figure 3; supplementary material, figure S3). The mitogenome dataset produced a well-resolved tree consistent with the UCE topology at the major clade level, though with minor ingroup rearrangements (figure 3; supplementary material, figure S4). Although divergence among major clades received high support, internal nodes within the Northern and Southern clades from the Americas were short and sometimes weakly supported, likely owing to low genetic variation among these samples.

**Figure 3.**
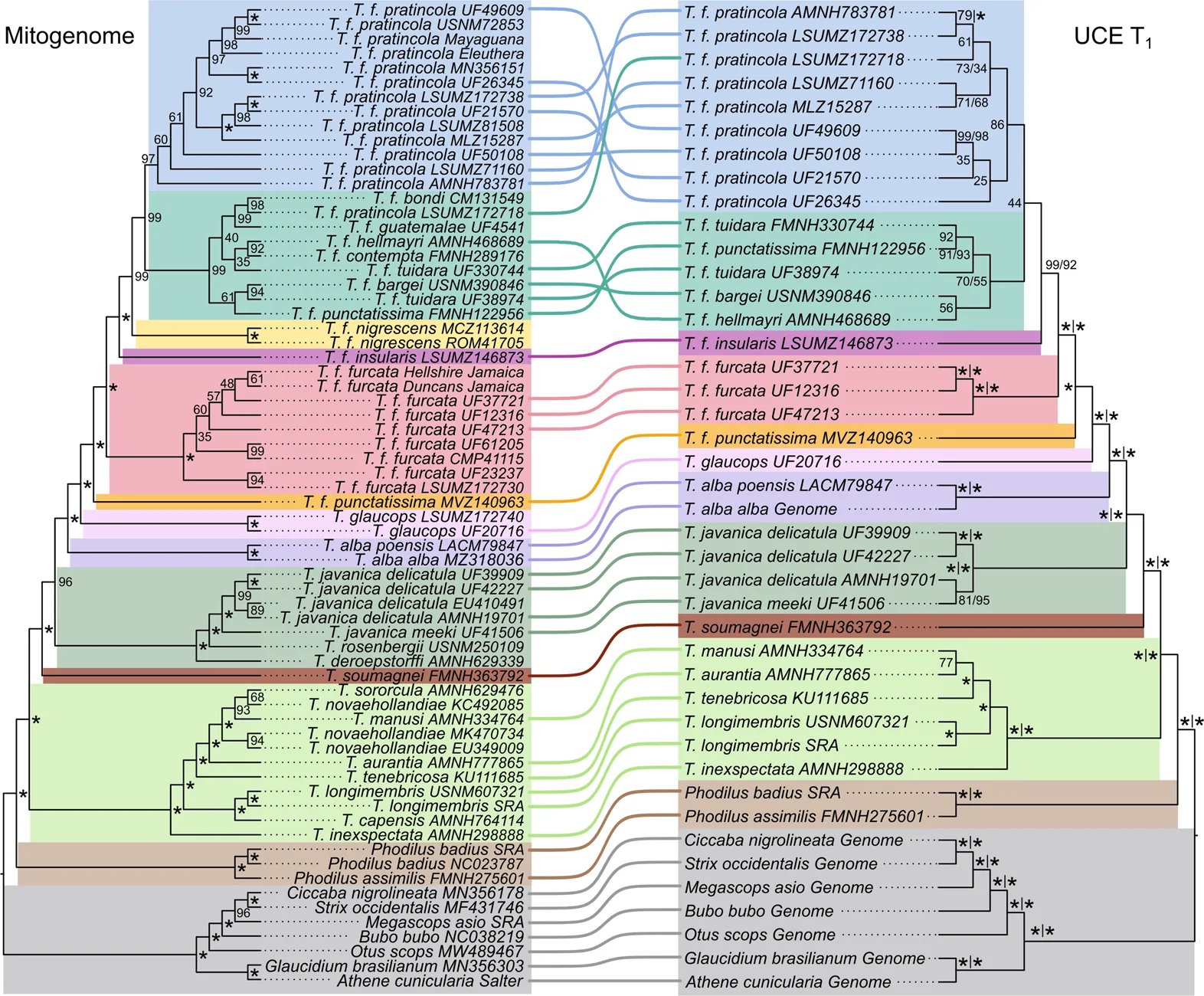
Topological comparison between the mitogenome and UCE (samples with >200 loci; T_1_) cladograms. Values near nodes are ultrafast bootstrap support, with an asterisk (*) denoting 100% support. For UCE T_1_, support values on the left side of the line are from the UCE tree T_0_ (used as the topological constraint), and those on the right side or a single value are from the constraint analysis.

Clades within *T. furcata* were largely congruent with current subspecies delimitation. Samples from continental North America, Bermuda, The Bahamas and Hispaniola formed the Northern clade (*T. f. pratincola*). The two *T. f. nigrescens* samples from Dominica*,* not represented in the UCE dataset, formed a clade distinct from the *T. f. insularis* sample from St. Vincent. Samples from Cuba, Jamaica and the Cayman Islands, representing *T. f. furcata*, also formed a monophyletic clade.

For the two Galápagos samples (in both the UCE and mitogenome trees), the Santa Cruz individual represented the earliest-diverging *T. furcata* lineage, while the San Cristóbal individual was nested within the Southern clade dominated by Central and South American subspecies. The San Cristóbal individual superficially resembles continental subspecies, whereas the Santa Cruz individual shows darker brown plumage and vermiculations on underparts that are typical of *T. f. punctatissima* (figure 4). A previous study [52] placed a Galápagos sample (from Isabela) as sister to *T. glaucops*, though with low support and limited loci. Our Southern clade also included several other island samples originally assigned to different subspecies based on geography. For example, samples from Curaçao (*T. f. bargei*) and Roatán (*T. f. bondi*) were grouped with continental South American samples with shallow divergences.

**Figure 4.**
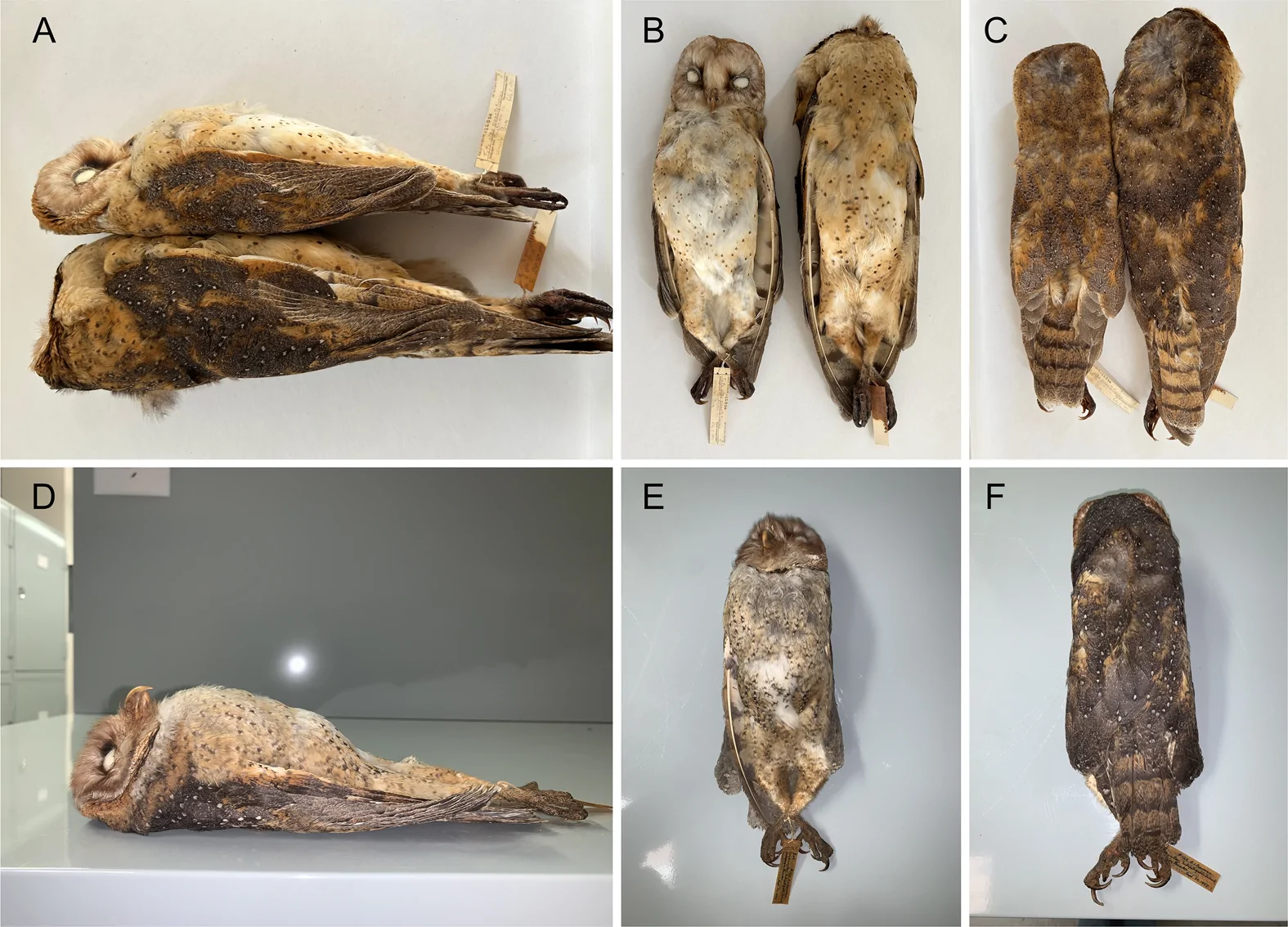
Photographs of Galápagos specimens from which our genetic samples were obtained. (A–C) San Cristóbal sample (FMNH 122956; male), shown on top in panel (A) and left in panels (B) and (C), along with a *Tyto furcata tuidara* specimen from Chile (FMNH 111644; female), bottom in panel (A) and right in panels (B) and (C). Note that the size difference between the two specimens may reflect reversed sexual dimorphism that is common in owls instead of solely geographical variation. (D–F) Santa Cruz sample with typical *T*. *f*. *punctatissima* plumage (MVZ 140963; female). Photo credits: John Bates (FMNH) and Carla Cicero (MVZ).

Species trees inferred using the good-quality samples (both ASTRAL and SVDquartets) recovered the same species-level topology as the ML trees (supplementary material, figure S5). Within *T. furcata*, the ASTRAL tree did not fully resolve the major clades identified in the ML analyses (supplementary material, figures S5 and S6), likely owing to the large number of historical samples, which typically have more missing data (supplementary material, table S3).

### (d) Divergence time and ancestral range reconstruction

Our results show that Tytonidae originated in the Oligocene (32.90 Ma; 95% HPD [30.90, 36.65]), and *Tyto* diverged from *Phodilus* during the Miocene (12.40 Ma; [10.23, 15.06]). Tytonidae today comprises two major clades: (i) the grass and masked owls, and (ii) the Red Owl of Madagascar, common barn owls and the Ashy-faced Owl of Hispaniola (figure 1). Western and Eastern Hemisphere *Tyto* diverged in the Pliocene (5.00 Ma; [3.75, 6.47]), with subsequent divergence estimates occurring in the Pleistocene.

For the global analysis, DEC was the best-fit model based on the Akaike information criterion (AIC; supplementary material, table S5). The most probable ancestral area for the most recent common ancestor (MRCA) of *Tyto* was estimated to be Africa + Eurasia + Oceania (figure 5A). Our results show that Western Hemisphere *Tyto* originated from Eurasia.

**Figure 5.**
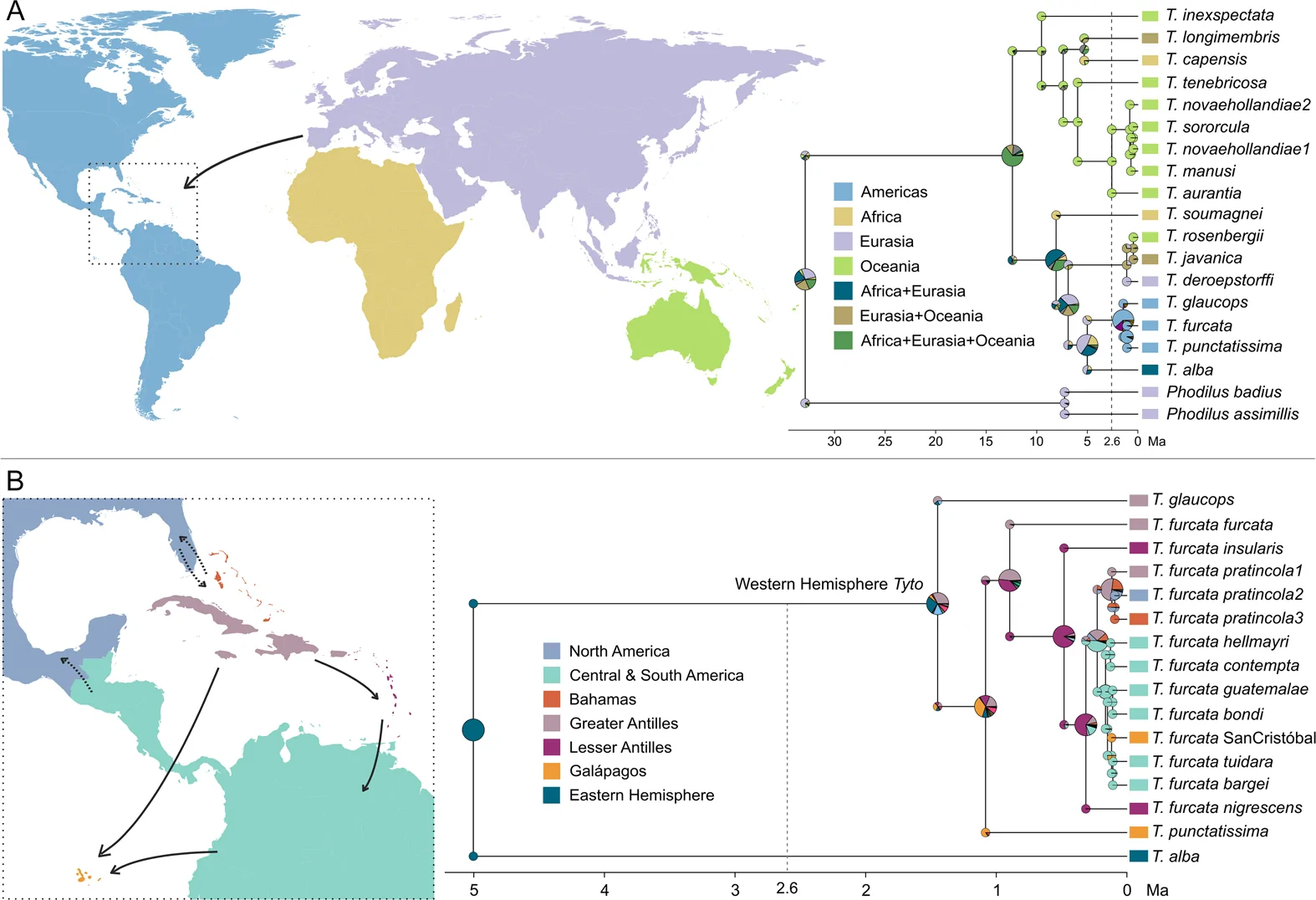
Ancestral range reconstructions using the time-calibrated mitogenome tree with a stratification at 2.6 Ma. (A) Analysis of Tytonidae based on four areas (Americas, Africa, Eurasia and Oceania). (B) Analysis of *T. alba* and Western Hemisphere *Tyto* based on seven areas (North America, Central and South America, The Bahamas, Greater Antilles, Lesser Antilles, Galápagos and Eastern Hemisphere). Arrows on the map depict potential colonization routes; dotted arrows denote more speculative pathways.

For the analysis focusing on Western Hemisphere *Tyto*, DIVALIKE+J was the best-fit model based on AIC (supplementary material, table S5). The MRCA of Western Hemisphere *Tyto* colonized the Caribbean, probably first through the Greater Antilles (figure 5B), and then the Lesser Antilles, before colonizing the continental Americas. Colonization from the Caribbean to the continental Americas shows more complex patterns owing to reverse colonization (e.g. from the North America continent to The Bahamas and Bermuda) and low phylogenetic resolution within the Northern clade (figure 2). We also recovered two independent colonization events to the Galápagos, one from the Greater Antilles to Santa Cruz Island, and a recent one from South America to San Cristóbal Island. Although the +J models provided the best statistical fit across our model comparisons, their biological interpretations should be treated with caution [53]. The main biogeographic patterns we discuss are consistent across models.

### (e) Quaternary *Tyto* extinctions

There are no known Quaternary *Tyto* species extinctions on continents. Many insular *Tyto* taxa became extinct worldwide during the Quaternary, though well-supported last occurrence dates for these taxa, and therefore, the causes of their extinctions remain largely unknown (supplementary material, table S6). Caribbean islands have experienced the greatest number of extinctions, particularly at the species level (N = 6). Associated with many of the extinct Caribbean *Tyto* were endemic mammals that are now also extinct. Different patterns appear in other island systems, for example, the Indo-Pacific islands show more recent losses, the majority of which may represent local extirpations (N = 4), whereas the Mediterranean exhibits more ancient extinctions during the Pleistocene (N = 3). However, we note that these patterns may partly reflect differences in the availability of fossils and comparative osteological material.

## 4. Discussion

We leveraged UCE and mitogenome data from modern, historical and ancient samples, including century-old museum specimens and material from fossil sites up to 296 years old, to elucidate Tytonidae phylogenetic relationships and colonization pathways. Our results support that *Tyto* originated in the Eastern Hemisphere and colonized the Western Hemisphere through the Caribbean, particularly through the Greater Antilles. From there, *Tyto* dispersed across the wider Caribbean, reached the Galápagos and spread to adjacent continental areas. The Caribbean was (and still is) the centre of *Tyto* diversity in the Western Hemisphere but is depauperate today compared with the past, likely owing to human arrivals to the islands beginning at least 6000 years ago. Even though many insular *Tyto* species are no longer extant, islands have acted as museums of diversity, sites of recent diversification and sources of continental diversity.

### (a) *Tyto* diversification across the Western Hemisphere

Our ancestral range reconstructions based on the extant taxa are consistent with previous work [51,52] in supporting an Eastern origin of Western Hemisphere *Tyto*. Both Uva *et al.* [52] and our study estimated a split between *T. alba* and Western Hemisphere *Tyto* at around 5 Ma. With comprehensive sampling across Caribbean islands, we identified a single colonization event from Eurasia to the Caribbean (Greater Antilles), followed by diversification across Caribbean islands. The relatively recent *Tyto* colonization of the Western Hemisphere is compatible with the fossil record. Pleistocene fossils of *T. furcata* are common and widespread on the North American continent [54], as is also the case with Caribbean *Tyto* taxa. In neither region have *Tyto* fossils been found in pre-Quaternary sites.

After *Tyto* arrived on Caribbean islands from the Eastern Hemisphere, these islands served as stepping stones for colonization of the continental Americas. Antillean *T. furcata* lineages (nominate race of Cuba, Jamaica and the Cayman Islands, and *nigrescens* and *insularis* of Lesser Antilles) exhibit deeper divergences than the two continental *T. furcata* clades, with notable osteological, plumage and morphometric differences [21,22]. By contrast, continental *T. furcata* lineages show minimal genetic divergence across latitude (Northern vs Southern clades), consistent with previous studies using microsatellites and mitochondrial data that found little to no genetic structure among continental populations [52,55]. The strong dispersal ability of *T. furcata* has likely promoted gene flow among the continental populations [55], as well as between the continents and immediately adjacent island populations (e.g. Curaçao, Roatán and Trinidad and Tobago). Our samples from the Bahamian archipelago did not form a monophyletic clade (figure 2), suggesting multiple colonization and/or reverse-colonization events between the archipelago and nearby North America or other Caribbean islands. The Bahamian archipelago comprises the northernmost Caribbean islands and has been strongly shaped by Pleistocene sea-level fluctuations [56]. These islands had expanded land area during glacial periods and reduced area during interglacials, although they were probably never completely submerged. These dynamics may have directly influenced patterns of *Tyto* dispersal and colonization.

Despite considerable effort, we did not recover adequate sequence data for extinct Caribbean giant *Tyto*, likely owing to degradation. Therefore, it remains unclear whether *T. pollens* and *T. ostologa* originated from a single or multiple colonization events. Suárez & Olson [24] speculated multiple colonization events based on morphological differences among Caribbean giant *Tyto* taxa, although the morphology of insular taxa can be exceedingly difficult to use to determine phylogenetic relationships [57,58]. In particular, island gigantism is a common phenomenon in birds [2,59], which often hinders phylogenetic placement (e.g. [60]).

Traditional island biogeography is based on the paradigm that continental species are the sources of island diversity (e.g. [61]). However, diversification patterns observed in both extant and extinct *Tyto* indicate that the Caribbean, rather than the nearby continents, was once a barn owl biodiversity hotspot, with multiple species, both giant and dwarf, inhabiting these islands (supplementary material, table S6). One lineage from this Caribbean radiation subsequently colonized the American continents. Therefore, our findings provide further evidence that islands are not sinks of continental diversity [62]. The island-to-continent pattern has been noted for several other Western Hemisphere lineages during the Cenozoic, such as *Turdus* thrushes [6], New World orioles [7], New World blackbirds [63], lizards [64,65] and bats [66,67], and is increasingly being recognized in other island systems, such as Wallacea [68].

### (b) Double colonization of the Galápagos

Unexpectedly, our findings reveal two independent colonization events of the Galápagos, one around the mid-Pleistocene and another in the late Quaternary. The recent colonization of San Cristóbal by ‘continental’ *T. furcata* is unsurprising given its closer proximity to the South American continent, extensive human disturbance and high number of non-native species (e.g. [69]). By contrast, barn owls from the other Galápagos islands (e.g. Santa Cruz, Isabela and Floreana) show differences in plumage (e.g. figure 4), osteology [22,23] and genomic data (herein) from other *T. furcata* lineages, supporting the recognition of *T. f. punctatissima* as a distinct species (*Tyto punctatissima*). Double or multiple independent colonization events of the Galápagos have been reported for rice rats, lizards and some insects [70], although they appear to be much rarer in birds.

### (c) Island extinction and conservation

Modern biodiversity and species distributions have been reshaped by millennia of human impacts, resulting in local extinctions, species invasions and the replacement of ecological specialists by synanthropic, human-tolerant generalists [71]. This replacement reduces local and regional diversity, eliminates distinctive taxa and drives widespread biotic homogenization [72], especially on islands [73,74]. The extinct Caribbean barn owls relied on diverse native mammalian prey, such as hutias, Lesser Antilles rice rats, West Indian spiny rats, West Indies shrews [22,24] and perhaps others. Humans arrived in the Caribbean during the mid-Holocene [75] and since have exploited the endemic fauna [76,77] and introduced exotic species [71–74]. These anthropogenic factors led to the extinction of many specialist species. The recent establishments of *T. furcata* on Hispaniola (circa mid-1900s [78]), Puerto Rico (first nesting record in 2015 [79]) and likely also San Cristóbal of Galápagos, as well as its rapid expansion across the continental Americas with minimal genetic divergence, suggest that *T. furcata*, as a human-tolerant generalist, can rapidly disperse and establish on both islands and continents. Once established, they may serve as sources for further colonization of neighbouring islands or adjacent continental regions, potentially outcompeting local species. The endemic *T. punctatissima* is already extirpated from Floreana of Galápagos along with numerous other taxa after humans colonized in the nineteenth century [23]. These results highlight the need for expedited studies on insular populations, especially those remaining of *T*. *punctatissima*.

Despite their synanthropic nature, many common barn owls are themselves in decline in parts of North America and Europe [80,81]. These declines may reflect shifts in human disturbance, as traditional, small-scale agricultural practices have given way to modern monocultures, pesticides and rodenticides [80,82]. This is exacerbated by more frequent extreme weather events [83] and vehicle strikes [84]. Caribbean barn owl lineages may be especially vulnerable as these islands are subject to drastic habitat alteration [85] and introduced mammalian predators (e.g. [86]). Efforts to increase knowledge of Caribbean owl populations can save the most evolutionarily distinct and range-restricted barn owl lineages in the Western Hemisphere.

## Supplementary Material



## Data Availability

Data and scripts that support the findings of this study are available in Figshare [87]. Data produced by this study (raw reads and annotated mitogenomes) have been deposited onto NCBI SRA (BioProject ID: PRJNA1242826) and GenBank (accessions: PX262149-PX262200). Supplementary material is available online [88].
