# Supplementary material for: Caribbean Islands facilitated the colonization of Western Hemisphere barn owls, yet experienced rampant extinction
Source: Proc Biol Sci. 2026 Oct 21;293(2081):20261022. doi: 10.1098/rspb.2026.1022 (PMC13682925; doi:10.1098/rspb.2026.1022)
Supplement: Supplementary file 1 [file rspb.2026.1022.zip › rspb.2026.1022/rspb20261022_si_001.pdf]

Supplementary Materials for:

Caribbean Islands facilitated the colonization of Western Hemisphere barn owls, yet experienced rampant extinction

Min Zhao[1,2,§], David W. Steadman[1], Michelle J. LeFebvre[1], Robert P. Guralnick[1], Joshua R. Doby[1], Julie M. Allen[2], Jessica A. Oswald[1,3,§]

1. Florida Museum of Natural History, University of Florida, Gainesville, FL 32611, USA
2. Department of Biological Sciences, Virginia Tech, Blacksburg, VA 24061, USA
3. U.S. Fish and Wildlife Service, National Fish and Wildlife Forensic Laboratory, Ashland, OR 97520, USA

§ corresponding author: oswaldj3@gmail.com; balaenazhao@gmail.com

Article DOI: 10.1098/rspb.2026.1022

This file contains Supplementary Information and Supplementary Figures S1-S6. Supplementary Tables S1-S6 can be found in the Excel spreadsheet "Supplementary_Tables.xlsx".

Table of Contents

Supplementary Figures **2**

Supplementary Information **9**

Radiocarbon dating 9

DNA extractions 9

Target enrichment protocol 9

Extracting mitogenomes using Nextflow/eager 10

Justification for fossil calibrations 10

References ***11***

Supplementary Figures

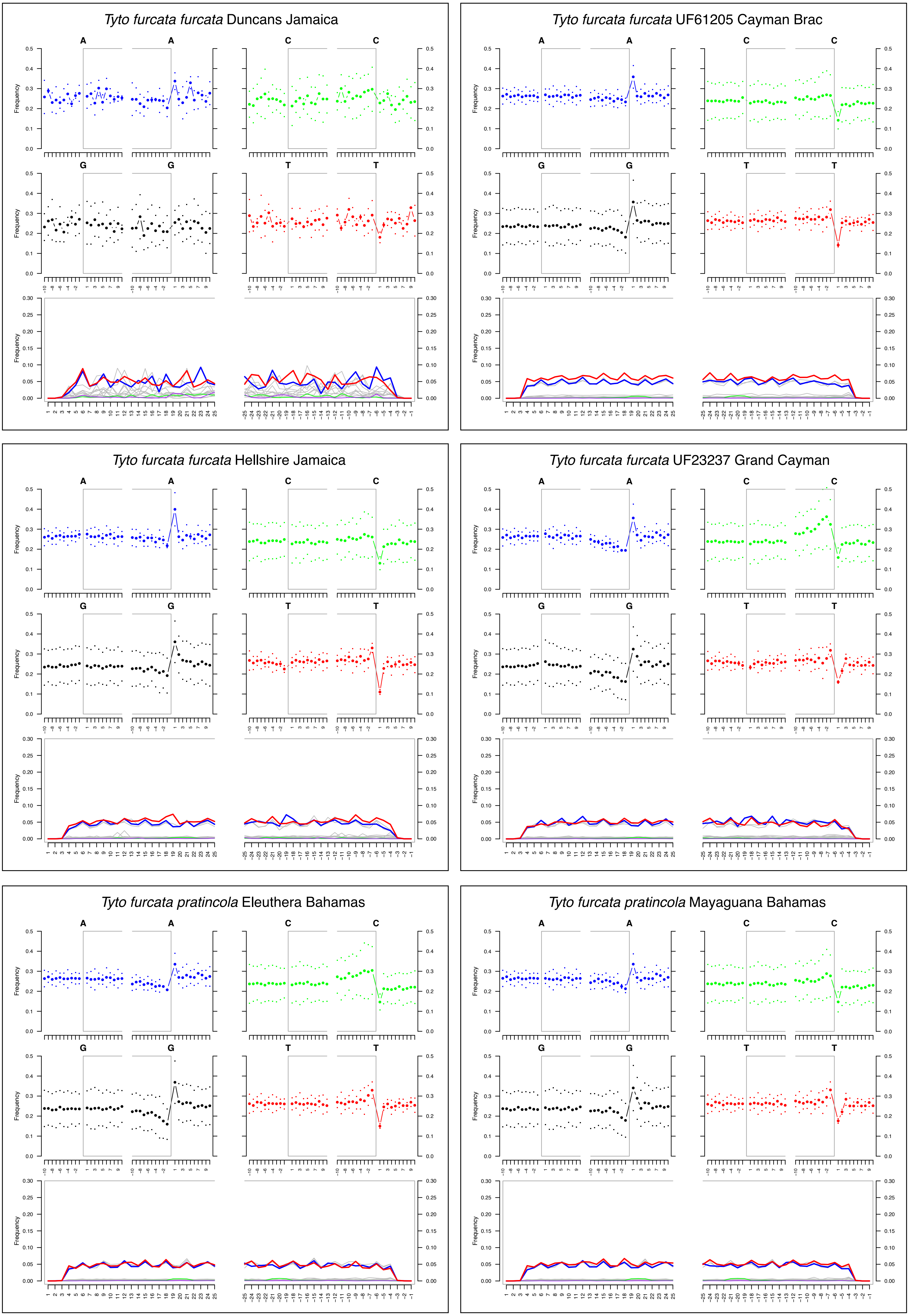

Figure S1. Misincorporation plots generated by mapDamage in the eager pipeline for the six fossil samples. None of these plots show the C->T or G->A substitutions typical of ancient DNA damage. Frequency for soft-clipped bases are not shown in the plots.

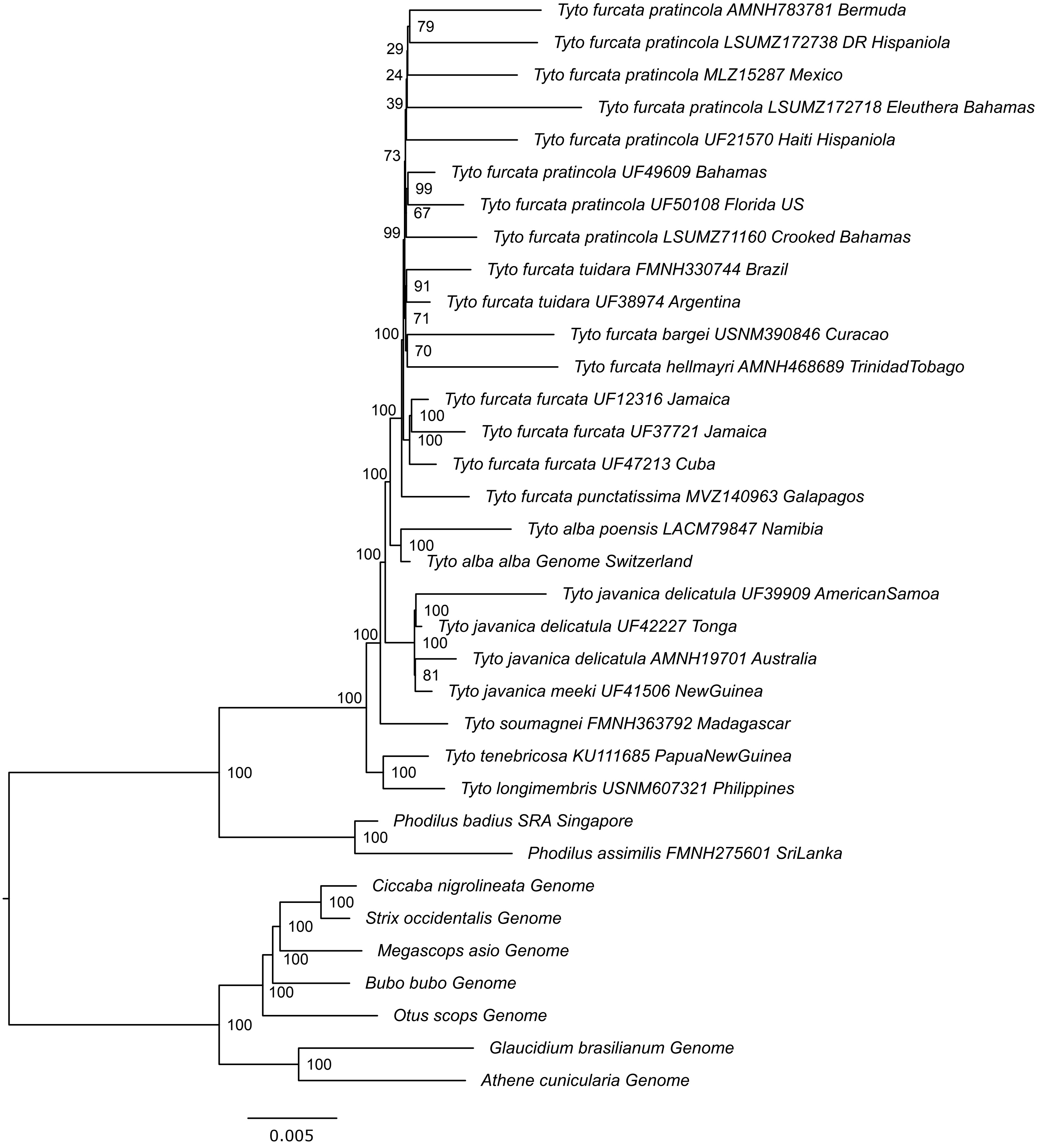

Figure S2. Concatenated UCE tree (T_0) using the dataset that includes samples with > 1500 loci, estimated by IQ-TREE2 under maximum likelihood. Support values at nodes are based on 1000 ultrafast bootstrap replicates.

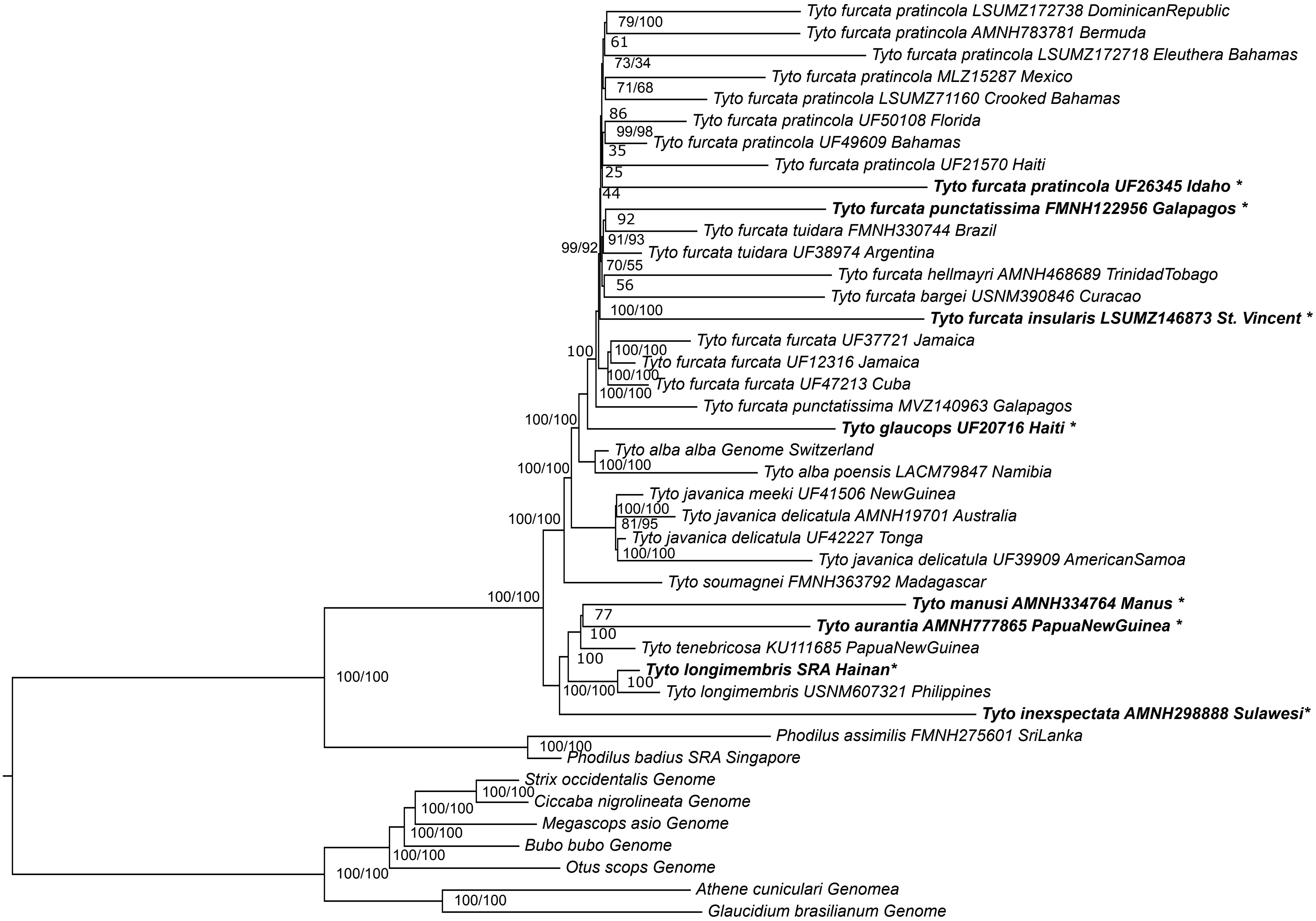

Figure S3. Concatenated UCE tree (T_1) using the dataset only including samples with > 200 loci, estimated with a topological constraint (T_0) in IQ-TREE2 under maxmimum likelihood. Values at nodes are ultrafast bootstrap support values (value before slash: support in T_0; value after slash or without a slash: support in T_1). Tip labels in bold (marked with *) are the samples with > 200 loci but < 1500 loci.

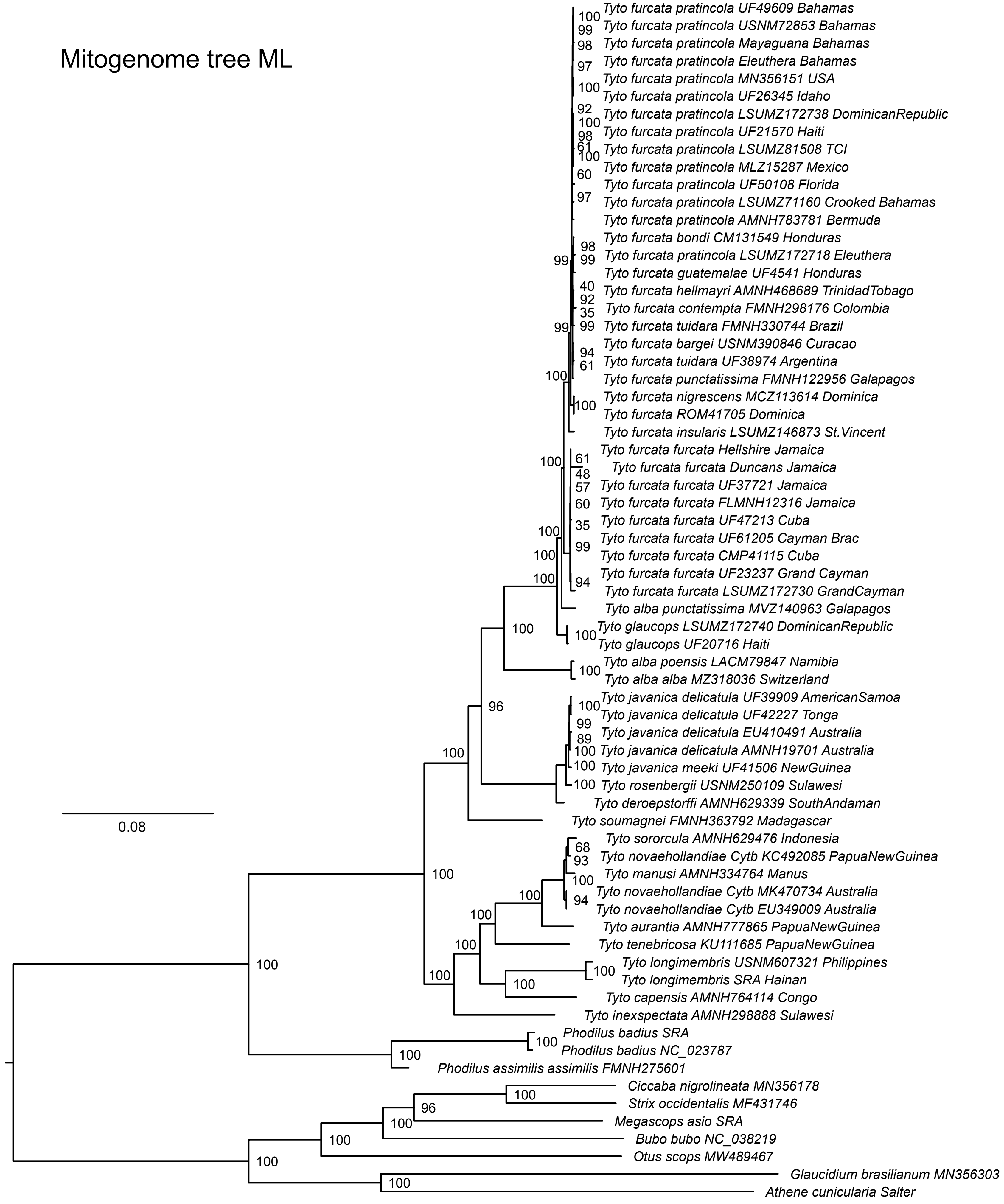

Figure S4. Mitogenome tree estimated by IQ-TREE2 under maximum likelihood. Support values at nodes are based on 1000 ultrafast bootstrap replicates.

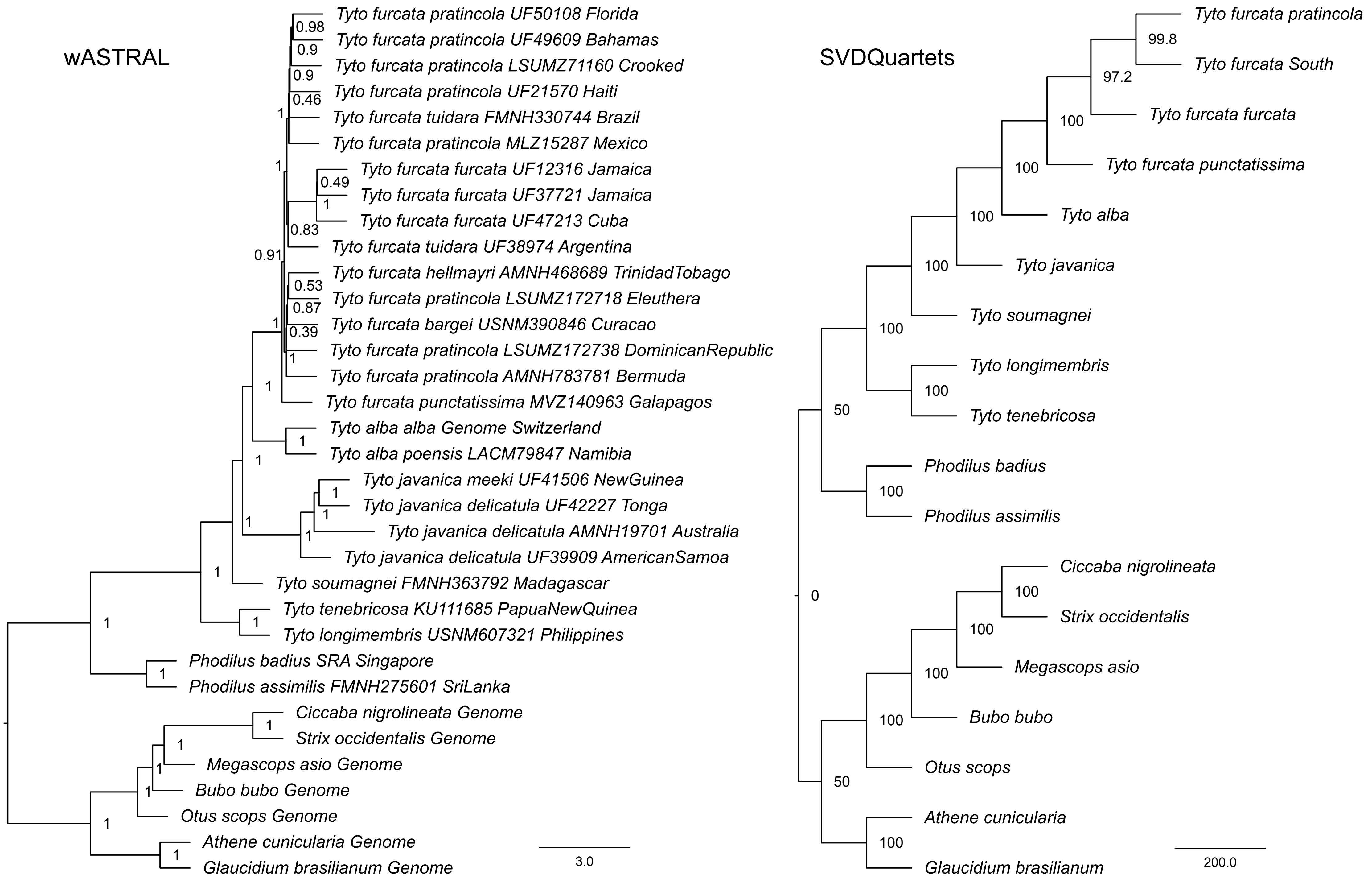

Figure S5. Species trees estimated using the UCE dataset with good-quality samples (> 1,500 loci). Two methods (weighted ASTRAL and SVDQuartets) were used.

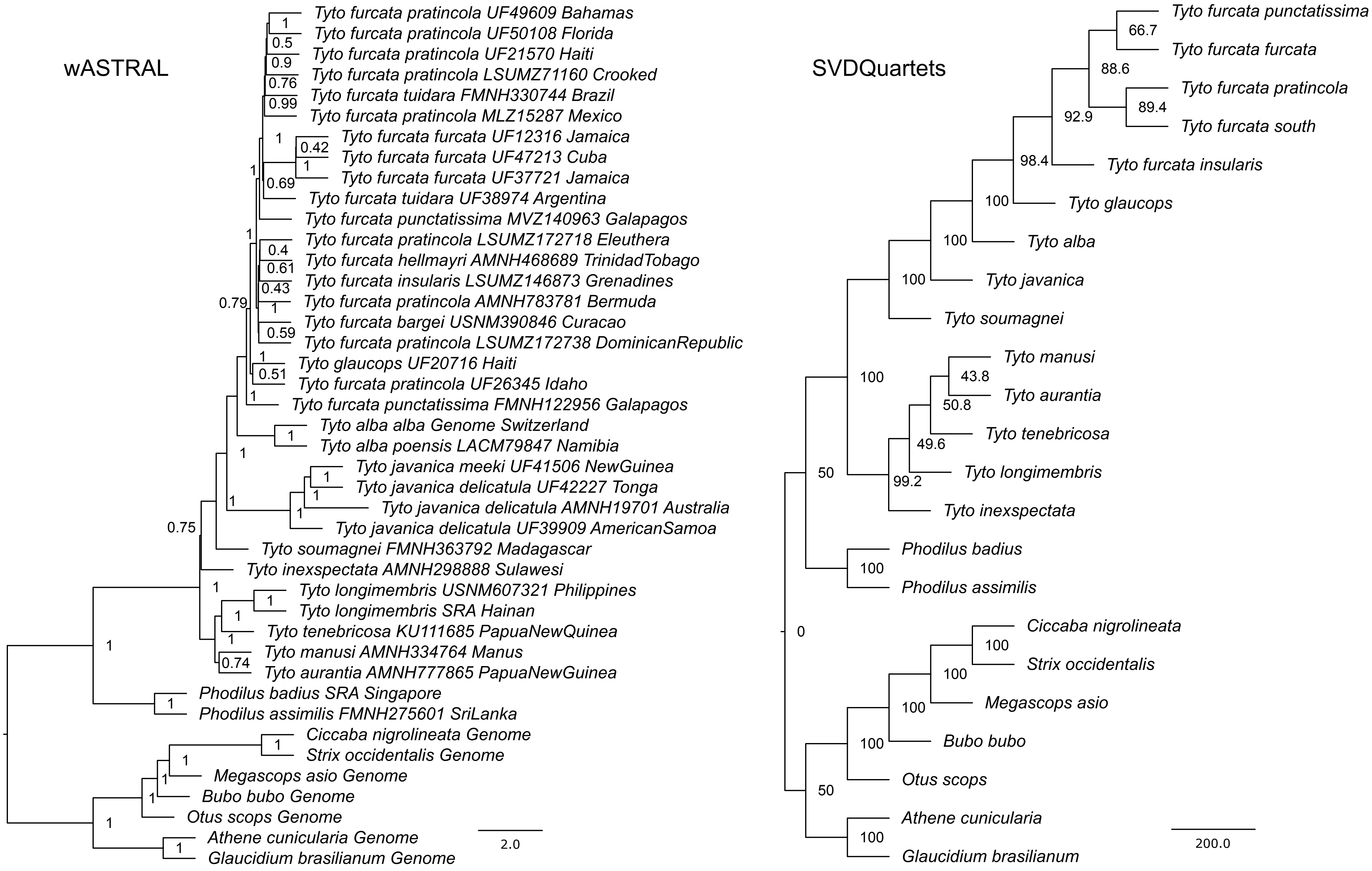

Figure S6. Species trees estimated using the UCE dataset with both good-quality samples (> 1,500 loci) and lower-quality samples (> 200 but < 1,500 loci). Two methods (weighted ASTRAL and SVDQuartets) were used.

Supplementary Information

Radiocarbon dating

Half of each fossil sample was used for ancient DNA (aDNA) extraction; the other half was sent to the University of Georgia Centre for Applied Isotope Studies (UGAMS) for radiocarbon dating (UGAMS pre-treatment methods are outlined at https://cais.uga.edu). Ultimately, some of these samples were modern in age, but we prepared DNA libraries for them as if they were ancient samples.

DNA extractions

All fossil samples were prepared in a lab dedicated to aDNA extractions at the University of Florida. Each fossil sample was soaked in a 5% bleach solution for five minutes to remove surface contaminants. The fossil was crushed in liquid nitrogen and combined with 949 µl of 0.5 M ethylenediaminetetraacetic acid (EDTA), 25 µl 20 mg/ml proteinase K, 21 µl of 10 mg/ml 1,4-dithiothreitol (DTT), and 5 µl of 10% sodium dodecyl sulfate (SDS). Samples were incubated at 60°C for 24 hours and intermittently vortexed. Samples then were concentrated with an Amicon Ultra-4 Centrifugal Filter Unit, purified using a MinElute PCR Purification Kit, and eluted in 48ul of EB buffer.

The toe pad samples were rinsed in EDTA (pH 8.0) then placed in an extraction buffer containing ATL (Qiagen), proteinase K, and 1 M DTT. The samples were incubated at 60°C for 24 hours and then the resultant lysed samples were cleaned and concentrated with a MinElute PCR Purification Kit.

Target enrichment protocol

DNA libraries were produced for fossils and ribs using an IDT xGen™ Methyl-Seq DNA Library Prep Kit following the standard protocol but excluding the bisulfite conversion step (which is only necessary to study methylation; per consultation with staff of Swift Biosciences [subsumed by IDT]). This kit is designed for highly damaged, fragmented DNA which is typical of historical and ancient DNA samples. DNA libraries for toe pads were produced with IDT xGen ssDNA & Low-Input DNA Library Prep Kit. DNA libraries were produced from tissue samples with an IDT xGen™ DNA Lib Prep EZ kit. The enzymatic shearing was set to 13 minutes to produce 350 bp fragment sizes according to manufacturer's guidelines. DNA concentration from all the above steps was quantified with a Qubit® 2.0 Fluorometer.

Modern (tissue) and historical (rib, toe pad) sample libraries were pooled according to similarity in age and library concentration of samples. A 1:250 ratio of mitochondrial baits (Arbor 303008.v5 developed based on *Tyto alba*; GenBank accession EU410491) was mixed with UCE baits (Arbor myBaits UCE Tetrapods 5Kv2) to increase the recovery of mitochondrial genomes and UCEs. The modern sample enrichment protocol followed that on the Arbor

303008.v5 kit. During amplification, 16 PCR cycles increased the overall concentration prior to sequencing. Bead clean ups were performed on the PCR products. DNA yield was quantified with a Qubit® 2.0 Fluorometer.

Genomic libraries from fossil samples were double-enriched following the myBaits high-sensitivity protocol (version 5) with mitochondrial baits (Arbor 303008.v5). The hybridization temperature was set to 60˚C; the sample was incubated for 12-16 hours per enrichment round. Two 50 µl PCR reactions were prepared for the entire resuspended DNA library (30 µl total volume). Using the entire sample maximizes yield and can also control for PCR duplicates. The samples were subjected to 16 PCR cycles. The DNA concentrations of the resultant PCR products were combined and quantified with a Qubit® 2.0 Fluorometer. The PCR product was concentrated with a vacuum centrifuge. The concentrated product then had a second round of enrichment following the protocol except for the PCR step. In this step, to further inhibit PCR duplicates, the two 50 µl PCR reactions for this sample were split into four 25 µl reactions. Following PCR, the products were combined, bead cleaned, and quantified with a Qubit® 2.0 Fluorometer. Libraries were quantified, cleaned, and pooled at the UF ICBR and sequenced on an Illumina NovaSeq, S4, lane using a 2x150 platform.

Extracting mitogenomes using Nextflow/eager

For fossil, toe pad, and rib samples, we trimmed an additional of 10 bases from the 3' end of R1 reads and 10 bases from the 5' end of R2 reads after regular adapter removal, following the manufacturer's recommendations to eliminate any synthetic cytosine methylation introduced during library preparation using AdapterRemoval v2 (Schubert et al., 2016). PCR duplicates were removed with MarkDuplicates.

For two species of Tytonidae (*Tyto longimembris* and *Phodilus badius*) and one species of Strigidae (*Megascops asio*) with published target enrichment reads in the NCBI SRA (see Table S1), we applied the previously described eager pipeline to extract mitogenomes from raw reads using mitogenomes of their closest relatives available as references.

Justification for fossil calibrations

The maximum age of crown Strigiformes is based on the earliest strigiform species reported, *Ogygoptynx wetmorei* (Vickers-Rich and Bohaska, 1976), from the North American Late Paleocene (Mammal Paleogene zones MP1, 61.7 Ma); it has both tytonid and strigid features, but is too old to be at the branching point of Tytonidae and Strigidae (Göhlich and Ballmann, 2013). The minimum age of crown Tytonidae is based on the first clearly tytonine species, the European *Prosybris antiqua*, possibly from the early Oligocene (MP23, 30.9 Ma; (Göhlich and Ballmann, 2013; Mlíkovský, 1998)). The maximum age of crown Tytonidae is based on the European *Necrobyas rossignoli* (MP 17B, 37.5 Ma; (Göhlich and Ballmann, 2013)), which was considered a stem group representative of Tytonidae (Mourer-Chauviré, 1987). We also used

37.5 Ma as the minimum age for crown Strigiformes. The minimum and maximum ages of crown Strigidae are more elusive, but Strigidae is considered younger than Tytonidae based on fossil records (Göhlich and Ballmann, 2013; Mayr, 2009). Because the oldest strigid fossils dated to the early Miocene of Europe (Mammal Neogene zones MN2; (Göhlich and Ballmann, 2013)), and there are no unambiguous Paleogene strigid fossils (Mayr, 2009), we tentatively used the upper and lower bounds of Mammal Neogene zones MN1 + MN2 (20.0 Ma – 23.03 Ma) for crown Strigidae.

References

Göhlich UB, Ballmann PA. 2013 A new barn owl (Aves: Strigiformes: Tytonidae) from the Middle Miocene of the Nördlinger Ries (Germany) with remarks on the history of the owls. In Paleornithological Research 2013 – Proceedings of the 8th International Meeting of the Society of Avian Paleontology and Evolution. (eds: Ursula B. Göhlich, Andreas Kroh) Naturhistorisches Museum Wien.

Mayr, G., 2009. Paleogene Fossil Birds. Springer Berlin Heidelberg, Berlin, Heidelberg. https://doi.org/10.1007/978-3-540-89628-9.

Mlíkovský, J., 1998. A new barn owl (Aves: Strigidae) from the early Miocene of Germany, with comments on the fossil history of the Tytoninae. J. Ornithol. 139, 247–261. https://doi.org/10.1007/BF01653335.

Mourer-Chauviré C. 1987 Les Strigiformes (Aves) des Phosphorites du Quercy (France): systématique, biostratigraphie et paléobiogéographie. In l'evolution des oiseaux d'apres le temoignage des fossiles, Table Ronde internationale du CNRS, Lyon-Villeurbanne, 18-21 Septembre 1985.

Schubert, M., Lindgreen, S., Orlando, L., 2016. AdapterRemoval v2: rapid adapter trimming, identification, and read merging. BMC Res. Notes 9, 88. https://doi.org/10.1186/s13104-016-1900-2.

Vickers-Rich, P., Bohaska, D.J., 1976. The World's Oldest Owl: A New Strigiform from the Paleocene of Southwestern Colorado, in: Olson, S.L. (Ed.), Collected Papers in Avian Paleontology Honoring the 90th Birthday of Alexander Wetmore, Smithsonian Contributions to Paleobiology. Smithsonian Institution Press, Washington, D.C., pp. 87–93. https://doi.org/10.5479/si.00810266.27.87.
